# Laminin-derived peptide drives the cardiomyogenic potential and cardiac cells functionality

**DOI:** 10.3389/fbioe.2025.1629412

**Published:** 2025-08-08

**Authors:** Simona Casarella, Federica Ferla, Dalila Di Francesco, Veronica Pagani, Carolina Di Varsavia, Irene Regano, Francesca Boccafoschi

**Affiliations:** ^1^Department of Health Sciences, University of Piemonte Orientale “A. Avogadro”, Novara, Italy; ^2^Laboratory for Biomaterials and Bioengineering, Canada Research Chair Tier I for the Innovation in Surgery, Department of Min-Met-Materials Engineering and Regenerative Medicine, CHU de Quebec Research Center, Laval University, Quebec City, QC, Canada

**Keywords:** laminin, cardiomyogenesis, integrins, cardiac tissue engineering, bioactive peptides

## Abstract

Laminin represents a major component of the basement membrane in cardiac tissue. Through integrins binding, laminin can sustain cell adhesion, proliferation, differentiation and mechanotransduction. The role of a small bioactive peptide (KKGSYNNIVVHV) derived from laminin in cardiac differentiation and functionality has been demonstrated. Briefly, results showed how the presence of laminin-derived peptide enhanced the differentiation of mesenchymal stem cells into cardiomyocytes-like phenotype, by modulating not only the cytoskeletal apparatus but also enhancing the cardiac and adhesion markers. Moreover, neonatal mouse cardiomyocytes in presence of the peptide showed well organized cytoskeletal rearrangements and regulated contractility. Therefore, in this study the role of laminin-derived peptide in guiding cardiomyogenesis has been characterized, opening innovative approaches in functional cardiac tissue engineering and regenerative medicine.

## 1 Introduction

Cardiovascular diseases, including heart failure after a myocardial infarction (MI), are a leading cause of death worldwide, with MI affecting roughly three million people worldwide (Jenča et al., 2021). In MI pathogenesis modifiable factors, such as lifestyle, and non-modifiable factors, such as age and genetics, are involved (Jenča et al., 2021). Atherosclerosis is a major cause of MI, often leading to vascular blockage and resulting in fatal outcomes in 7 out of 10 cases. Moreover, the rupture of atherosclerotic plaques triggers inflammation and damages the myocardium, which hardly regenerates, leading to irreversible cardiac fibrosis (Zaman and Kovoor, 2014). Current clinical treatments for MI fall short in fully restoring heart function. Therefore, the optimal therapeutic goal for post-infarction treatment would be to reduce fibrosis and to promote the regeneration of myocardial tissue by stimulating the repopulation of the damaged tissue with functional contractile cardiomyocytes. In fact, fibroblasts and myofibroblasts play a key role in the formation of scar tissue in the injured area. Targeting these cells for therapeutic intervention represents an attractive strategy to minimize fibrosis and enhance functional tissue regeneration in the heart. By driving their activity, it may be possible to promote tissue regeneration and improve cardiac function following MI (Jenča et al., 2021; Mei and Cheng, 2020; Li et al., 2021; Talman and Ruskoaho, 2016).

Interestingly, over the past decade, the fields of biomaterials and nanotechnology have introduced innovative strategies for tissue regeneration using natural bioactive and biomimetic matrices, which can properly mimic the extracellular matrix (ECM) (Mei and Cheng, 2020; Li et al., 2021).

Actually, the ECM’s biochemical compositions and physical properties are crucial for tissue regeneration, with integrins and focal adhesions (FAs) playing pivotal roles (Santoro et al., 2019). FAs are complexes that link the cells to the ECM structural proteins, also facilitating cell movement. FAs show different molecular compositions, which may vary with respect to the surrounding environment. A common feature is that FAs are linked by actin stress fibers and include transmembrane integrin receptors, specifically heterodimers of integrins α and β, which directly bind the intracellular environment to the ECM (Santoro et al., 2019; Geiger and Bershadsky, 2001). Integrins are involved in multiple functions, including organogenesis, gene expression regulation, proliferation, differentiation, migration, and cell death. Through the activation of several pathways such as Focal Adhesion Kinase (FAK), Rho-associated coiled-coil-containing protein kinases (ROCKs), and mitogen-activated protein kinases (MAPKs), integrins act on cardiac myocytes, inducing and maintaining their differentiation through hypertrophic and anabolic responses (Geiger and Bershadsky, 2001; Ross, 2004; Ross and Borg, 2001; Kovacic-Milivojević et al., 2001).

In adult cardiac cells, the major expressed integrins are α1β1, α5β1, and α7β1, which respectively bind collagen, fibronectin, and laminin. Studies have shown that integrin α7β1, specifically binding to laminin, has a protective effect on cardiomyocytes and is reduced after MI, highlighting the importance in enhancing cardiac function. Moreover, it has been proved that modulating ECM through laminin content could provide structural and functional support to the cardiomyocytes stiffness (Geiger and Bershadsky, 2001; Ross and Borg, 2001; Okada et al., 2013). Therefore, optimization of the recognition of key integrins in guiding cell differentiation and cell survival could be exploited in cardiac tissue engineering in order to restore a functional cardiac tissue after an MI. In this context, novel peptides identified in ECM proteins are being explored for their potential in biomaterials, therapeutics and tissue engineering, enhancing cell adhesions, cell-matrix interactions and signaling pathways. For instance, synthetic extracellular matrices functionalized with bioactive peptides, such as the Arg-Gly-Asp (RGD) sequence, derived from laminin, have been extensively employed to enhance cell adhesion, proliferation, and differentiation in various tissue engineering applications. The inclusion of RGD peptides into biomaterials has been shown to improve reendothelialization of cardiovascular devices and promote bone tissue regeneration (Boateng et al., 2005; Wang et al., 2019). Thus, the use of ECM-related peptides to enhance the surface properties of tissue engineering and biomedical devices presents a solid, versatile, and convenient approach.

In this work, the role of laminin-derived active peptide, identified by Urushibata et al. (KKGSYNNIVVHV) (Urushibata et al., 2010), applied to cardiac regeneration and functional restoration has been characterized. Interestingly, Urushibata et al. have identified, among different active sequences related to the laminin globular domains (LG) of the α2 chain, the KKGSYNNIVVHV small sequence present in LG1, which should bind selectively to the integrin α7β1. Thus, we demonstrated the cardiomyogenic properties of the laminin-derived peptide in driving the cardiac differentiation of hMSC-Y201 (James et al., 2015; Galarza Torre et al., 2018) and the capability in regulating cytoskeletal and contractility properties in primary cardiomyocytes.

## 2 Materials and methods

### 2.1 Peptide coating

In order to evaluate the effect of the laminin-derived active peptide on cell adhesion and cell differentiation, the KKGSYNNIVVHV (1.36 kDa) (Urushibata et al., 2010) (from here following indicated as G2) (Sigma Aldrich, Milano, Italy) peptide has been used. The peptide was diluted at a stock concentration of 1 mg/mL with phosphate-buffered saline (PBS) and absorbed overnight in cell culture plates at 4°C. Then washed with phosphate-buffered saline. Experiments were performed with the peptide coating concentrations: 100 μg/mL and 50 μg/mL (Urushibata et al., 2010; Sung et al., 2023; Li et al., 2014; Vroemen et al., 2025).

### 2.2 Cell culture

All *ex vivo* studies were approved by the Animal Experimentation Ethics Committee, University of Eastern Piedmont, Italy (DB064.N.TMC.). BALB/c mice were purchased by Charles River (Italy).

#### 2.2.1 Cardiomyocytes-like differentiation of immortalized human mesenchymal stem cell hMSC-Y201

Human bone marrow mesenchymal stem cells (Y201 BM-MSCs) (James et al., 2015) (LGC standard, Milan, Italy) were maintained at 37°C in humidified atmosphere (5% CO2) and cultured in Dulbecco’s Modified Eagle Medium (DMEM) Low Glucose (Euroclone, Milan, Italy) supplemented with 15% (v/v) fetal bovine serum (FBS), penicillin (100U/mL), streptomycin (0.1 mg/mL), amphotericin (0.25 μg/mL) and L-Glutamine (2 mM) (all products from Euroclone, Milano, Italy). Y201 cells were seeded at a density of 1 × 10^3^ cells/cm^2^ in 6-well coated plates with the G2 peptide. The differentiation of MSCs into a cardiomyocytes-like cells was induced using a differentiation medium 1 consisting in DMEM Low Glucose (Euroclone, Milan, Italy) containing 10% FBS, followed by the addition of 5 µM 5-Azacytidine (Sigma Aldrich, Milano, Italy) and 1 µM retinoic acid (RA) (Sigma Aldrich, Milano, Italy), for the first 24 h. After 1 day, the medium was changed by adding differentiation medium 2: DMEM Low Glucose (Euroclone, Milan, Italy) enriched with 10% FBS, 10 ng/mL Transforming Growth Factor beta (TGF-β1), 10 ng/mL Insulin Growth Factor-1 (IGF-1) and 1 µM retinoic acid (RA) (Sigma Aldrich, Milano, Italy) (Sokolowska et al., 2020; Antonitsis et al., 2007; Gwak et al., 2009; Lv et al., 2018; Deng et al., 2016). Anti-integrin *β*1 (Bio-Techne, Italy) was used as neutralizing antibody and added to the medium at a final concentration of 500 ng/mL. The medium was renewed every 48 h for 7 days in order to properly maintain the differentiative/neutralizing effect.

#### 2.2.2 Primary cardiomyocytes isolation and culture

The isolation of mice neonatal ventricular cardiomyocytes (CM) has been optimized on the basis of two different protocols (Ravi et al., 2021; Pereira et al., 2021). Briefly, CM were isolated from the heart of 1–3 days-old mice. After separating the atria, the ventricula were minced and subjected to eight cycles of enzymatic digestion using collagenase type II (Sigma, Italy). The enzymatic cycles consisted of constant shaking at 180 rpm for 10 min at 37°C. The digested tissues were resuspended in 1X ADS buffer (11.64 mM NaCl, 0.54 mM KCl, 1.83 mM HEPES sodium salt, 0.08 mM NaH2PO4, 0.56 mM Glucose, 0.04 mM MgSO4 x 7 H2O) and transferred to the percoll gradient solution. After centrifugation at 1,500 rpm for 45 min, the ring on the percoll gradient was collected and incubated in tissue culture plates coated with G2 peptide with CM plating medium (Dulbecco’s Modified Eagle Medium (DMEM) high glucose, supplemented with 5% (v/v) fetal bovine serum (FBS), 10% (v/v) horse serum (HS), penicillin (100U/mL), streptomycin (0.1 mg/mL), amphotericin (0.25 μg/mL) and L-Glutamine (2 mM). Once the CM attached to the plate (after 48 h), the plating medium was changed with the growth medium (DMEM high glucose, supplemented with 2% (v/v) fetal bovine serum (FBS), penicillin (100U/mL), streptomycin (0.1 mg/mL), amphotericin (0.25 μg/mL) and L-Glutamine (2 mM) (All products were purchased from Sigma, Italy).

### 2.3 Fluorescence analysis

Cells were seeded onto pre-coated (50 μg/mL and 100 μg/mL of G2) glass coverslips as described before. At each time point, cells were fixed with 4% formalin in PBS (pH 7.4), rinsed, permeabilized with 0.1% TritonX100 in PBS (Sigma Aldrich, Milano, Italy), saturated with 5% Goat Serum (Euroclone, Milano, Italy) and blocked with 5% bovine serum albumin (BSA) (Sigma Aldrich, Milan, Italy). Samples were then incubated overnight at 4°C with primary antibodies anti-vinculin, anti-integrin *α*7 and anti-*α*-Actinin (all products from Sigma Aldrich, Milan, Italy) diluted at 1:100 in 2% Goat Serum (Euroclone, Milano, Italy), 1% BSA (Sigma Aldrich, Milan, Italy) and 0.1% TritonX (Sigma Aldrich, Milano, Italy) in PBS. Vinculin was revealed by secondary Texas Red-labeled anti-mouse IgG antibodies (1:500; Vector TI 2000, Vector Laboratories, CA, United States). Phalloidin (Sigma Aldrich, Milan, Italy) staining was performed to reveal the presence of stress fibers and with 300 nM 4′,6-Diamidine-2-phenylindol dihydrochloride or 2-(4-Amidinophenyl)-6-indolcarbamidin -dihydrochloride (DAPI) (Sigma Aldrich, Milan, Italy) for the nuclei dye. A final rinsing was done before the samples were placed using a mounting medium (60% glycerol in PBS). In order to evaluate the stress fibers and focal adhesions’ formation and to analyze vinculin expression, representative images were taken with the fluorescence Leica DM2500 microscope (Wetzlar, Germany) and acquired *via* Leica software.

#### 2.3.1 Integrin *α*7 fluorescence quantification

In order to quantify the fluorescence, microscopy images were analyzed using ImageJ software 1.52r (ImageJ, United States). Single cells were manually selected as regions of interest (ROIs). Background fluorescence was measured from black regions. Area, mean gray value, and integrated density were selected from each image and data were analyzed to calculate the corrected total cell fluorescence (CTCF) using the formula:

CTCF = Integrated Density – (Area of selected cell X Mean fluorescence of background readings).

CTCF values were used to compare fluorescence intensity in CM and G2 groups and the data obtained were reported as a whisker plot.

### 2.4 Morphometrical analysis

Characterization of focal adhesions (FA) was evaluated by measuring the length (Buskermolen et al., 2018) and the total number of FAs per cell. In particular, the length of individual FA was measured from images at ×100 magnification with immersion oil by drawing the longitudinal axis of vinculin positive signal in at least 100 cells collected in 15 different microscope fields (DM2500 Leica microscope; 100x). The total number of cell’s focal adhesions was analyzed in at least 100 cells from 10 representative fields collected at ×20 magnification (DM2500 Leica microscope; 20x). All data were analyzed using ImageJ software 1.52r (ImageJ, United States). The normal distribution of the values (length and total number of FA) was reported as a Gaussian function.

### 2.5 Total RNA isolation and qrt-PCR

In order to evaluate the expression of cardiomyogenic-related genes, qRT-PCR was performed on GATA-4 and Troponin I. The relative expressions for the target genes were represented as fold change (2^−ΔΔCt^ method) with respect to the housekeeping gene GAPDH and normalized with respect to the control. The experiments were repeated in triplicate. Primary cardiomyocytes were maintained in culture with G2 (50 μg/mL) peptide coating for 10 days after the isolation, Y201 cells were cultured for 7 days.

#### 2.5.1 Total RNA extraction

Collection of total RNA extraction was performed at the end of each time point using TRIzol (Thermo Fisher Scientific, Milano, Italy). RNA purification was performed according to the manufacturer’s protocol (Thermo Fisher Scientific, Milano, Italy). The concentration and the purity of the extracted RNA were determined through the absorbance reading at 260 and 280 nm, using Nanodrop (Thermo Fisher Scientific, Milano, Italy). Afterward, DNAse treatment (DNAse I, Fermentas, St. Leon-Rot, Germany) was performed in order to digest single- and double-stranded DNA to oligodeoxyribonucleosides containing a 5′-phosphate.

#### 2.5.2 RNA retro-transcription

cDNA was synthetized from 1 µg of extracted RNA according to SensiFAST™ cDNA Synthesis Kit (Meridian Bioscience, United States) using the oligo (dT) primers.

#### 2.5.3 Real-time quantitative PCR

qRT-PCR was performed in 10 μL reaction volume containing 1 μL of reverse transcriptase products, 5 μL SensiFAST™ SYBR No-ROX kit (Meridian Bioscience, United States) and 10 µM each forward and reverse primers, as indicated in Supplementary Tables 1, 2 (see Supplementary Materials). Strips were placed in CFX Connect Thermal Cycler (BioRad, Hercules, CA, United States) with a reaction cycle of 95 °C for 3 min, followed by 39 cycles of 53°C for 30 s and anneal–extend step for 30 s at 65°C. Results were exported in CFX Maestro Software (BioRad, Hercules, CA, United States) and analyzed in Excel (Microsoft, Redmond, WA, United States). All experiments were performed in triplicate.

### 2.6 Protein expression

Western blot was performed on Y201 and CM cells seeded onto coated plates with G2 (50 μg/mL).

#### 2.6.1 Total lysis

After 7 days of culture for Y201 and after 10 days for CM, cells were lysed in boiling SDS (Tris-HCl 1 M pH 7,4, SDS 10%, PBS pH 7.4, ultrapure water), and cellular lysates were collected and stored at −20°C. Protein concentration was determined using Pierce BCA Protein Assay Kit (Thermo Fisher, Milan, Italy) according to the producer’s protocol. Samples were prepared for electrophoresis by dissolving in Laemmli Sample Buffer (62.5 mM Tris-HCl pH 6.8, 10% glycerol, 5% beta-mercaptoethanol, 0.005% bromophenol blue, 2% SDS) (Sigma Aldrich, Milano, Italy). Electrophoresis was performed using Sodium Dodecyl Sulphate-PolyAcrylamide Gel (SDS-PAGE) using 7.5% N, N′-methylenebisacrylamide (acrylamide) and then electrophoretically transferred to a nitrocellulose membrane (Amersham Biosciences, Little Chalfont, UK). Blotted proteins were blocked with 5% non-fat dried milk on PBS for 1 h at room temperature, then incubated overnight at 4°C with mouse anti-vinculin, rabbit anti-integrin *α*7, rabbit anti-integrin *β*1, rabbit anti-Connexin-43, rabbit anti-Troponin I, mouse anti-GAPDH, and mouse anti-Tubulin primary antibodies (all products from Abcam, Cambridge, UK) at a ratio of 1:1,000. After washing, membranes were incubated with HRP-conjugated secondary antibody (1:2000; Perkin-Elmer, Milan, Italy) for 1 h at room temperature, and bands were visualized using a chemosensitive visualizer (ChemiDocTM Touch Imaging System, Bio-Rad, Milano, Italy). Densitometric analysis was performed using ImageLab (BioRad, Hercules, CA, United States) software. Experiments were performed in triplicate.

#### 2.6.2 Subcellular fraction

After 7 days, Y201cells were lysed in subcellular fractionation buffer (10 mM Tris-HCl, pH 8, 1.5 mM MgCl_2_, 5 mM KCl, 50 μg/mL leupeptin, 5 μg/mL pepstatin A, 2 mM phenylmethylsulfonylfluoride), for 20 min on ice. Samples were mechanically disrupted with a manual Potter homogenizer (VWR, Milano, Italy) and centrifuged at 250,00 × *g* for 45 min. After the centrifuge, the soluble cytoplasmic (supernatant) and insoluble membrane (pellet) fractions were then recovered. The membrane fraction was then resuspended in boiling SDS (Tris-HCl 1 M pH 7,4, SDS 10%, PBS pH 7.4, ultrapure water). Cell lysates were prepared for electrophoresis by dissolving in Laemmli Sample Buffer (62.5 mM Tris-HCl pH 6.8, 10% glycerol, 5% beta-mercaptoethanol, 0.005% bromophenol blue, 2% SDS) (Sigma Aldrich, Milano, Italy) and analyzed directly by Western blot after SDS-PAGE separation as previously described. Experiments were performed in triplicate.

### 2.7 Video recording and contraction analysis

Cardiomyocyte contractility was recorded using a brightfield Optical Microscopy. Samples were visualized at ×40 magnification for cluster analysis. High-speed videos were captured at 60 frame rates of 60 s using a camera attached to the microscope ocular with a universal adapter. Videos were analyzed using the MYOCYTER macro in ImageJ software 1.52r (ImageJ, United States) following protocol described by Grune et al. (Grune et al., 2019). Briefly, the software identifies contracting regions based on pixel intensity changes between successive frames. Beating frequency was determined by counting contraction peaks over the recording duration, while contraction amplitude was evaluated by change between the relaxed and contracted states. Moreover, the peak time user indicates the time when the peak contraction occurs (how long it takes to reach the peak of the contraction, expressed in seconds). The systolic and diastolic interval (Systole user and Diastole user, expressed in seconds) show instead, respectively, the duration of the systole (time the cells are contracting) and the duration of the diastole (time the cells are relaxing).

### 2.8 Statistical analysis

All data were expressed as mean values ±standard deviation. Student’s t-test test performed on the software “Quantitative Skills SISA” was used for evaluating the significance of the results obtained. p-value was calculated and the differences between variables with a value of *p* ≤ 0.05 were considered statistically significant (*p ≤ 0.05) with respect to the control. Each experiment was repeated in triplicate.

## 3 Results

### 3.1 The role of laminin-derived peptide in cardiac differentiation of hMSC-Y201

In order to evaluate the effect of G2 peptide on cell adhesion and morphology, two concentrations were used: 50 μg/mL and 100 μg/mL.

**FIGURE 1:**
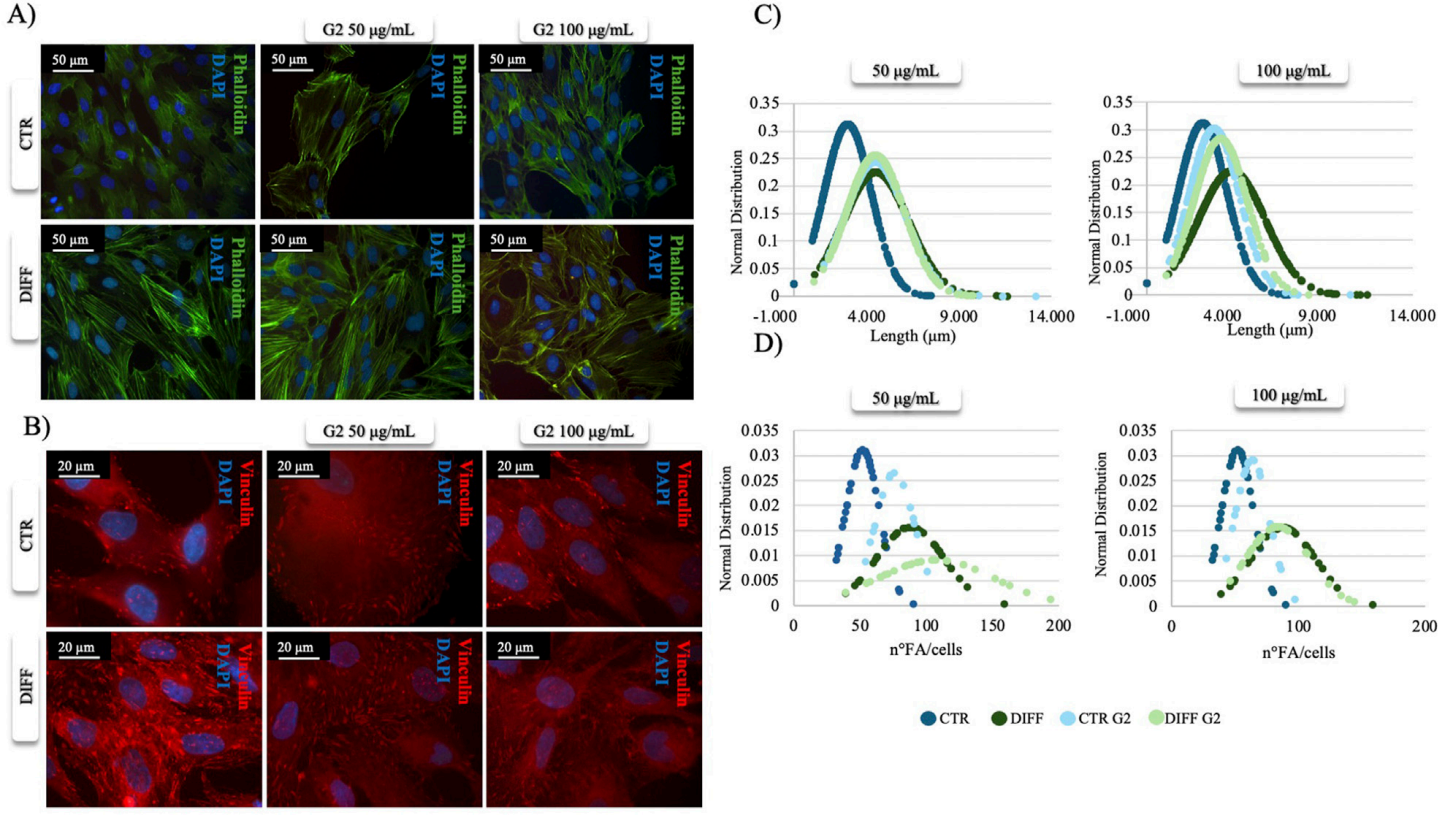
Morphometrical evaluation. **(A)** Cell morphology obtained after Phalloidin (green) staining and **(B)** anti-Vinculin (red) staining, nuclei were stained with DAPI (blue). Images represent control and differentiated cells, seeded onto G2 coated surfaces. Images are representative of all results obtained in three independent experiments. Scale bar: 50 μm (phalloidin staining), 20 μm (vinculin staining). **(C,D)** morphometric analyses of focal adhesion length (μm) and focal adhesion number of MSCs treated with different concentrations of G2, in control and differentiative medium. Data are represented as Gaussian curves with n ≥ 100. Measures were obtained from fluorescence microscopy images. Results are significant with respect to control, p ≤ 0.05.

After 7 days of culturing, phalloidin staining revealed morphological changes in the presence of peptide coating (Figure 1A). In fact, in the presence of G2, an enhanced and organized structure of stress fibers was observed not only in differentiated cells but also under control conditions treated with the peptide, suggesting a role for G2 in controlling cytoskeletal arrangements toward a cardiac muscle phenotype. The effect was more relevant using 50 μg/mL. Moreover, vinculin staining revealed how, at the same concentration (50 μg/mL), FA showed an enhanced and more organized expression in both differentiated and control conditions when in presence of G2 peptide (Figure 1B). Indeed, morphometrical analysis related to FA length and number per cell (Figures 1C,D) showed that in presence of both concentrations of G2, the curves shift to the right, indicating an enhancement in terms of length and number of FA/cell in the differentiated cells compared to the respective control.

**FIGURE 2:**
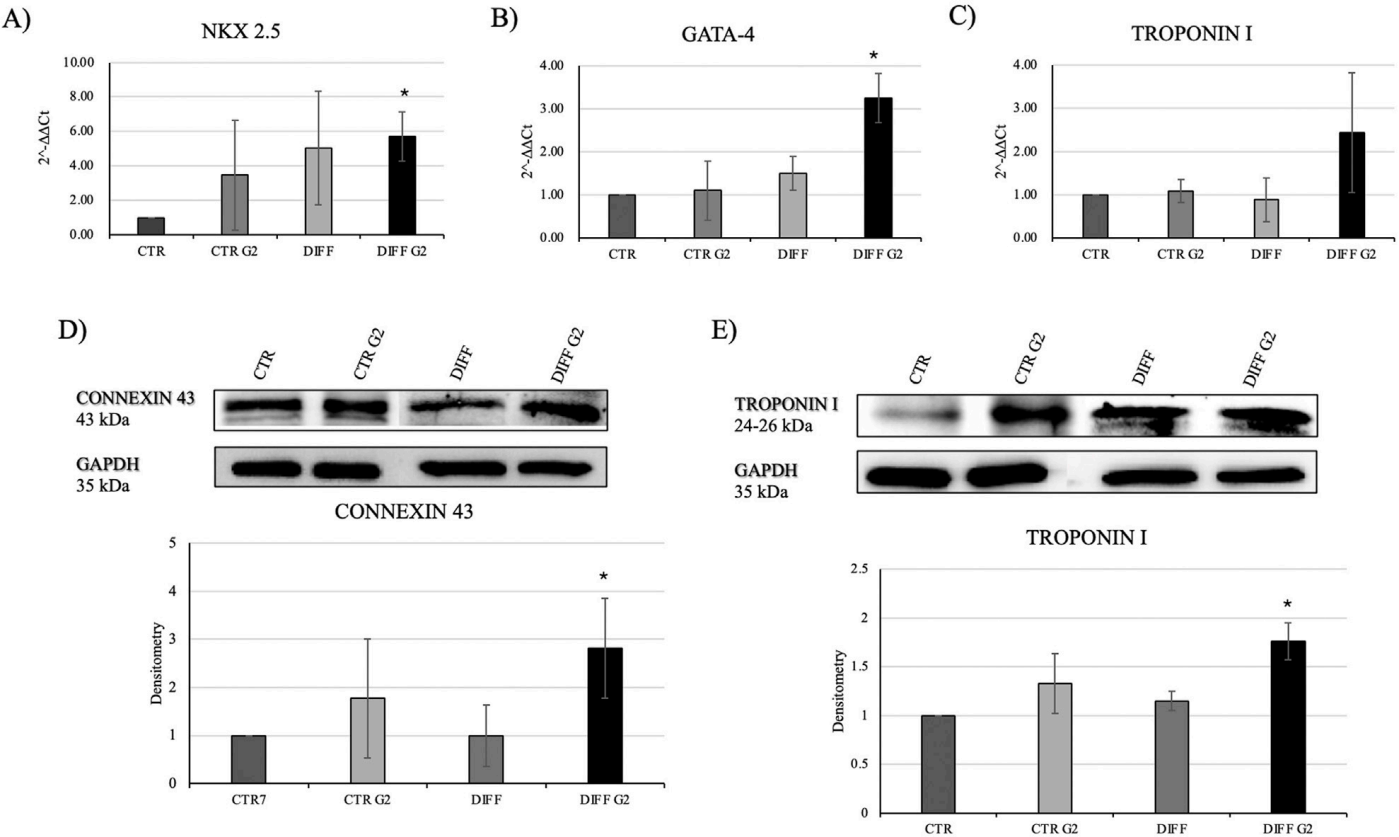
Evaluation of cardiac differentiation markers. Gene expression profiling of **(A)** NKX 2.5, **(B)** GATA-4 and **(C)** Troponin I. All results were normalized with respect to the control (CTR). Western blot analyses with **(D)** anti-Connexin 43 and **(E)** anti-Troponin I. GAPDH expression was used to normalize the results. Proteins were revealed on total lysates and all results were normalized with respect to control. Densitometry obtained represents results from three different experiments, expressed as mean ± SD. *Statistical significance with respect to control with p ≤ 0.05.

Once evaluated the initial cellular morphology and FAs involvement in hMSC-Y201, the lower concentration of the peptide enhanced not only the FAs involvement in differentiated cells, but also increased the organization of stress fibers, compared to the non-treated and treated cells with higher concentration of G2. Thus, 50 μg/mL concentration of G2 has been used for further analysis in cardiac differentiation. Considering these results, then two cardiac differentiation marker genes GATA-4 and NKX 2.5 have been evaluated (Figures 2A,B), and after 7 days of differentiation, in presence of the G2, the expression was enhanced. Moreover, Figures 2C,E show how both gene and protein expression of cardiac troponin I seemed to be enhanced after 7 days of differentiation and in presence of the peptide, in respect to the other conditions, suggesting an influence on cytoskeletal rearrangement. Nevertheless, to further analyze the cardiomyogenic potential, connexin 43 expression level was evaluated (Figure 2D). Western blot analyses revealed a significant enhancement of the connexin 43 in cells treated with the G2 compared to the control and differentiated cells not cultured on the peptide, highlighting the pivotal influence of G2 on cardiac behavior.

**FIGURE 3:**
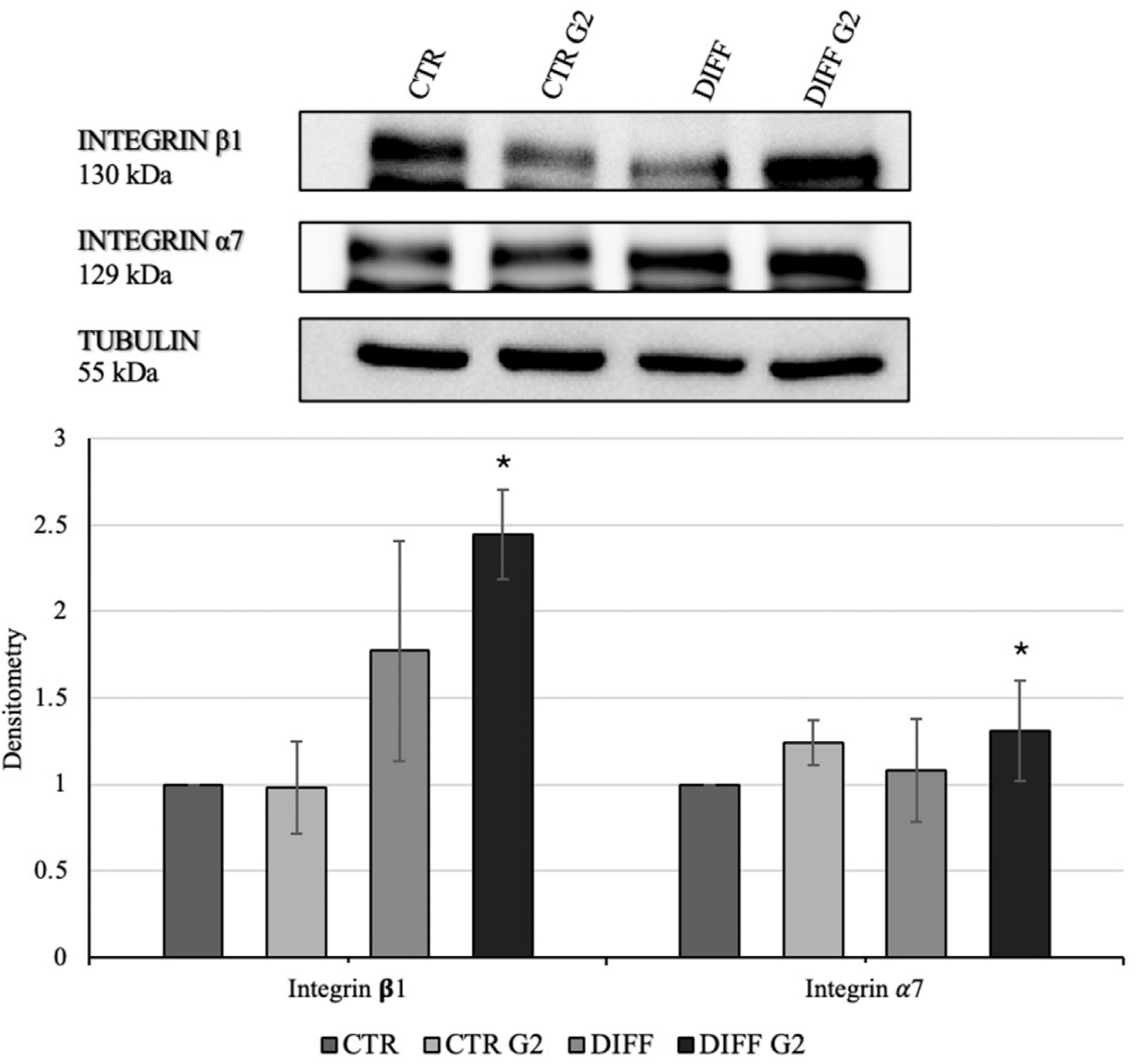
Western blot analyses with anti-Integrin *β*1 and anti-Integrin *α*7 antibodies. Tubulin expression was used to normalize the results. Proteins were revealed on total lysates and all results were normalized with respect to control. Densitometry obtained represents results from three different experiments, expressed as mean ± SD. *Statistical significance with respect to control was indicated with p ≤ 0.05.

**FIGURE 4:**
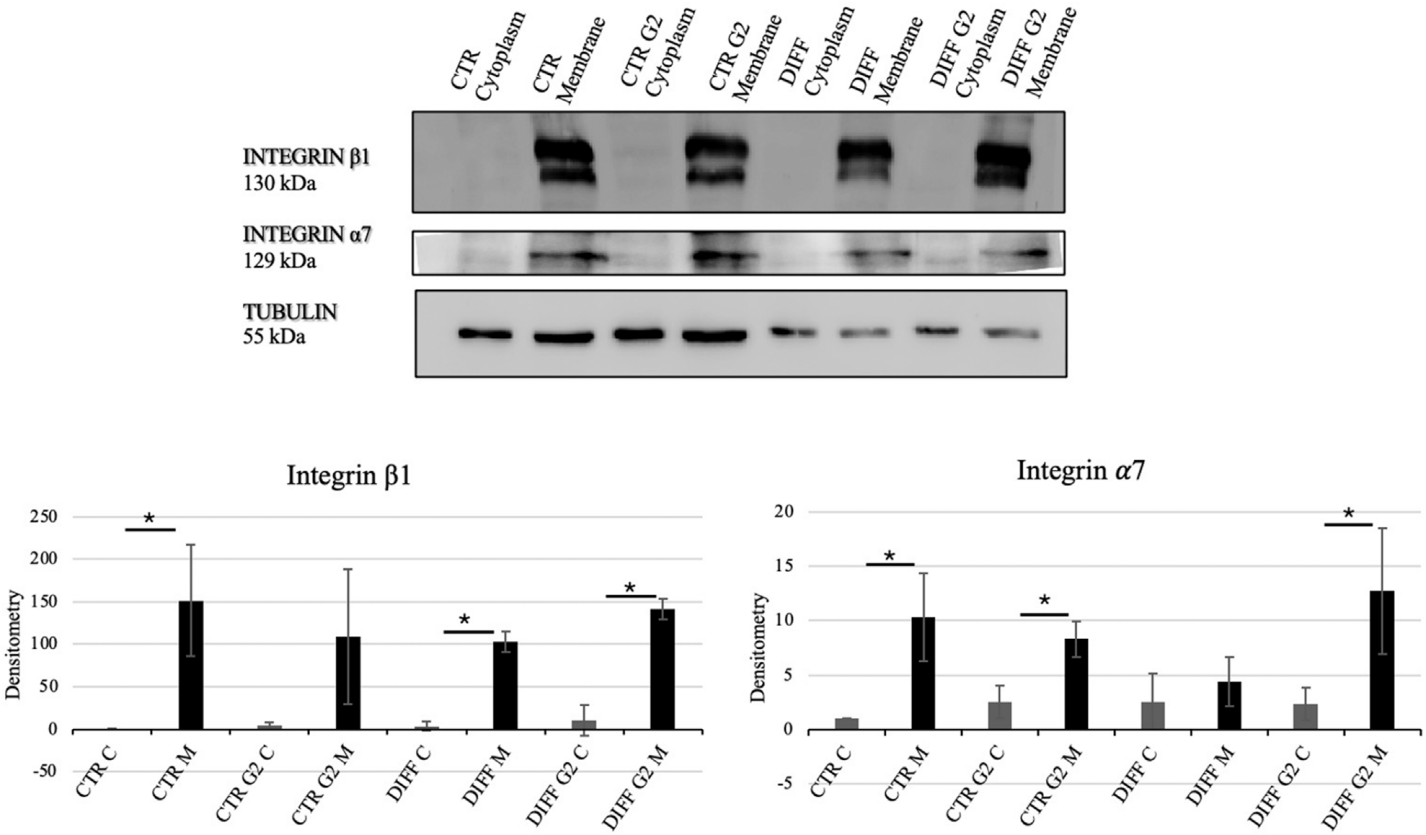
Western blot analyses using anti-Integrin *β*1 and anti-Integrin *α*7 antibodies. Tubulin expression was used to normalize the results. Proteins were revealed on fractionated lysates and all results were normalized with respect to control cytoplasmic portion. C=Cytoplasm, M = Membrane. Densitometry obtained represents results from three different experiments, expressed as mean ± SD. *Statistical significance with respect to the relative cytoplasmic portion with p ≤ 0.05.

In order to assess the interaction between the laminin-derived peptide G2 with the integrin *α*7*β*1, Western blot analyses for the two specific integrin subunits were performed. In presence of the peptide, differentiated cells showed a significantly enhanced expression of both integrin *α*7 and integrin *β*1 (Figure 3). Moreover, the subcellular expression of these two subunits revealed an enhancement not only in the membrane but also in the cytoplasmic fraction of the integrin *α*7 in the peptide-treated cells (Figure 4).

**FIGURE 5:**
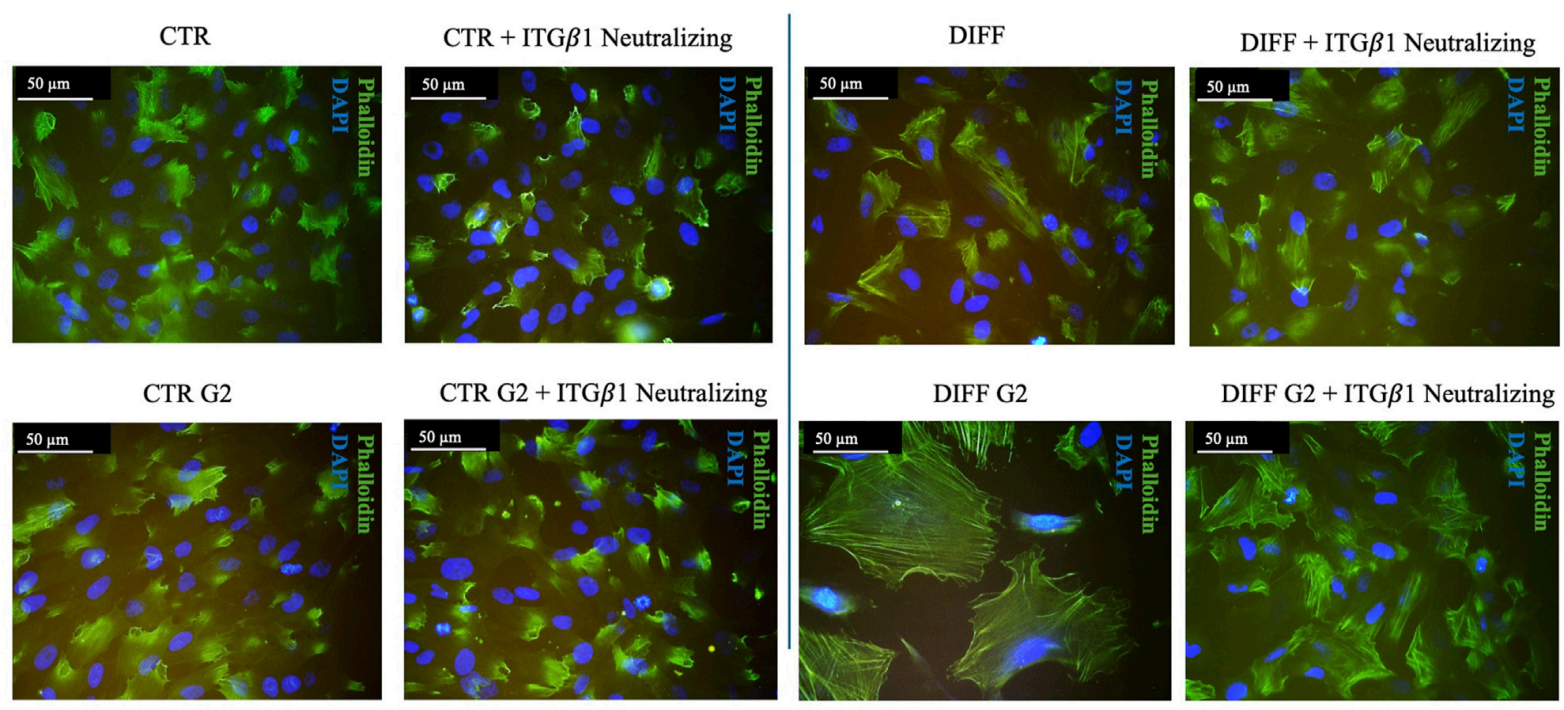
Morphological evaluation of Y201 cells after 7 days of culture with anti-integrin *β*1 as a neutralizing antibody. Cell morphology obtained after Phalloidin (green) staining, nuclei were stained with DAPI (blue). Images represent control and differentiated cells, previously seeded onto peptide coating surfaces. Images are representative of all results obtained in the three different experiments. Scale bar 50 μm.

**FIGURE 6:**
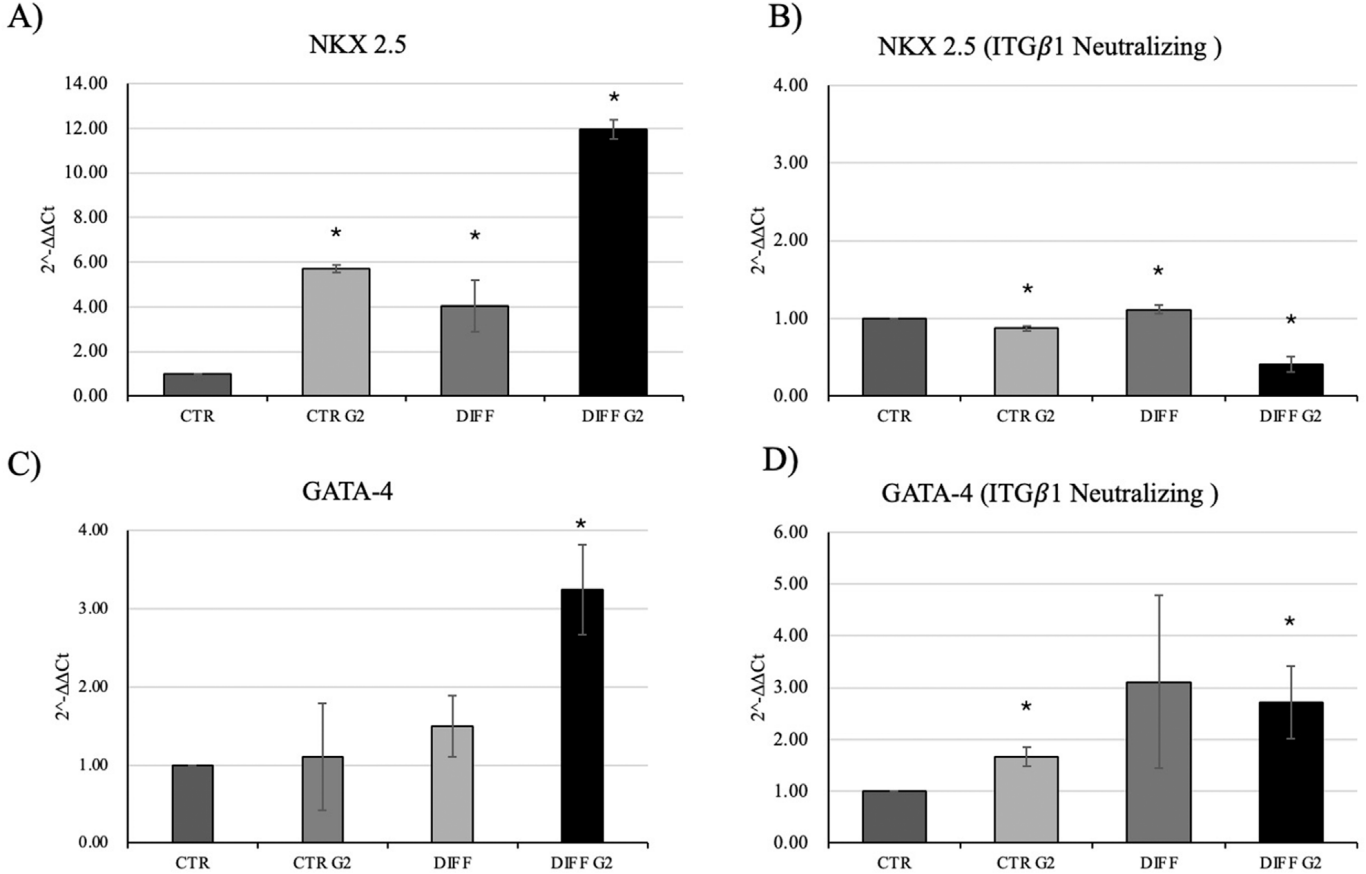
Gene expression profiling of NKX 2.5 and GATA-4 in **(A–C)** normal condition and with **(B–D)** anti-integrin *β*1 as a neutralizing antibody. All results were normalized with respect to the control (CTR). Results obtained from three different experiments, expressed as mean ± SD. *Statistical significance with respect to control with p ≤ 0.05.

Intriguingly, Figure 5 shows that, when in presence of the neutralizing anti-integrin *β*1 at 7 days, differentiated cells, seeded onto G2, loose the well-organized cytoskeletal structure, thus, the integrin blocking significantly interferes with cardiac differentiation in terms of contractile apparatus (Figure 5). Furthermore, qRT-PCR showed that NKX 2.5 (Figures 6A,B) was downregulated in presence of the neutralizing anti-integrin *β*1, confirming the cardiomyogenic role of the integrin *α*7*β*1-G2 peptide interaction. Moreover, the expression of GATA-4 (Figures 6C,D) in differentiated cells treated with G2 and neutralizing anti-integrin *β*1 resulted downregulated compared to cells subjected to the same treatment without the neutralization.

### 3.2 The role of G2 in cardiac phenotype maintenance and cytoskeletal rearrangements of primary neonatal cardiomyocytes

**FIGURE 7:**
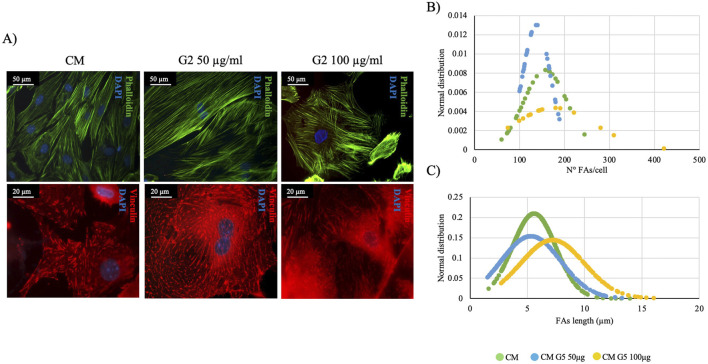
Morphometrical evaluation. **(A)** Cell morphology obtained after Phalloidin (green) staining anti-Vinculin (red) staining, nuclei were stained with DAPI (blue). Images represent control and treated cells with G2. Images are representative of all results obtained in the three different experiments. Scale bar: 50 μm (phalloidin staining), 20 μm (vinculin staining). **(B,C)** morphometric analyses of focal adhesion length (μm) and focal adhesion number of primary cardiomyocytes treated with different concentrations of G2. Data are represented as Gaussian curves with n ≥ 100. Measures were taken from fluorescence microscopy images. Results are significant with respect to control (CM), p ≤ 0.05.

Morphological analyses revealed how G2 peptide at 50 μg/mL do not alter the stress fibers formation and organization, as well as the presence of FA with anti-vinculin staining, similar to the control condition. On the contrary, at 100 μg/mL stress fibers and FA seem to be less organized with respect to the control (Figure 7A).

In fact, morphometrical analyses on FA length and number per cell (Figures 7B,C) demonstrated how in both evaluations, the curves of G2 at 50 μg/mL do not shift compared to the control, showing how the peptide, at lower concentration, could have a significant role in maintaining the cardiac phenotype. Thus, only 50 μg/mL has been used for further experiments.

**FIGURE 8:**
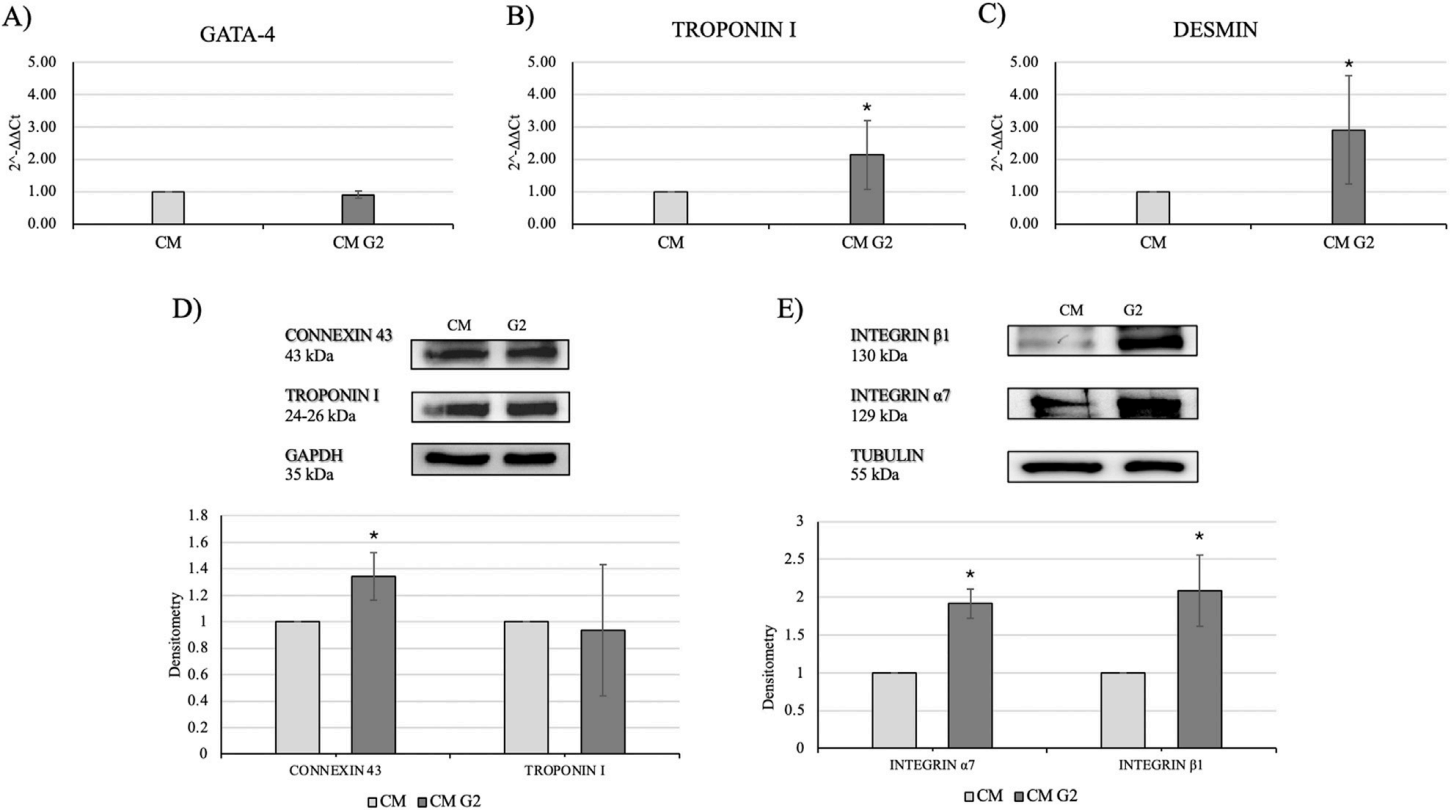
Evaluation of cardiac differentiation markers. Gene expression profiling of **(A)** GATA-4, **(B)** Troponin I and **(C)** Desmin. All results were normalized with respect to the control (CTR). Western blot analyses with **(D)** anti-Connexin 43 and anti-Troponin I antibodies and **(E)** anti-Integrin *β*1 and anti-Integrin *α*7 antibodies. The GAPDH and Tubulin expressions was used to normalize the results. Proteins were revealed on total lysates and all results were normalized with respect to control and differentiated not treated. Densitometry obtained represents results from three different experiments, expressed as mean ± SD. *Statistical significance with respect to control with p ≤ 0.05.

In order to assess the role of G2 on primary cardiomyocytes, cardiac differentiation marker GATA-4 was evaluated, and gene expression seemed not modified compared to the control. (Figure 8A). Nonetheless, gene expression of cardiac troponin I and desmin resulted significantly enhanced in presence of G2, indicating an active role in cytoskeletal regulation of cardiomyocytes (Figures 8B,C).

Moreover, Western blot analyses showed a slight but higher expression of connexin 43 in treated cardiomyocytes, as well as an enhanced expression of the two subunits of integrin *α*7 and *β*1, highlighting the role of G2-integrin complex (Figures 8D,E).

**FIGURE 9:**
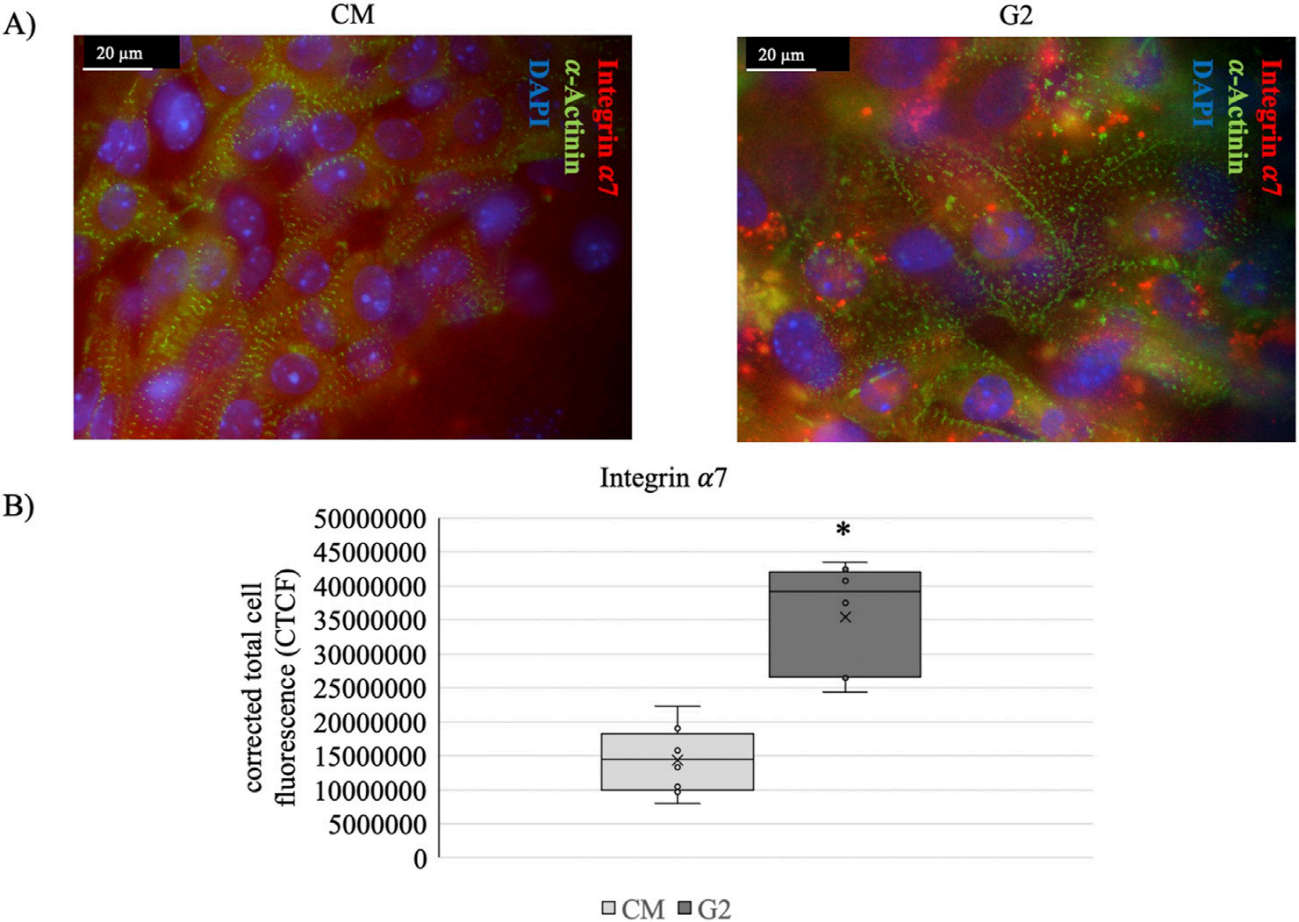
**(A)** Costameres formation revealed with anti-*α*-Actinin (green), anti-Integrin *α*7 (red) staining on cardiomyocytes (CM) and CM seeded onto G2 coating (G2). Nuclei were stained with DAPI (blue). Scale bar: 20 μm. **(B)** anti-Integrin *α*7 fluorescence quantification. The corrected total cell fluorescence (CTCF) is represented as whisker plot with n = 8. *Statistical significance with respect to CM with p ≤ 0.05.

To further confirm the role of the peptide in guiding laminin-mediated cytoskeletal rearrangements, staining of *α*-actinin and integrin *α*7 has been performed (Figure 9A). Figure 9B shows how in presence of G2 the expression of integrin *α*7 is enhanced, highlighting the role of G2 in guiding the integrin *α*7*β*1 recruitment, as well as, supporting the contractile cytoskeletal phenotype.

Given the enhanced expression of connexin 43 and the costameric organization, contractility analyses were performed using MYOCTER macro in ImageJ software. Supplementary Videos 1S, 2S show respectively the control and the G2 conditions. It is clear that the presence of G2 enhances the clustering formation with respect to the control. Furthermore, videos show how the presence of G2 may influence not only clustering of the cells, but also the contraction force.

**FIGURE 10:**
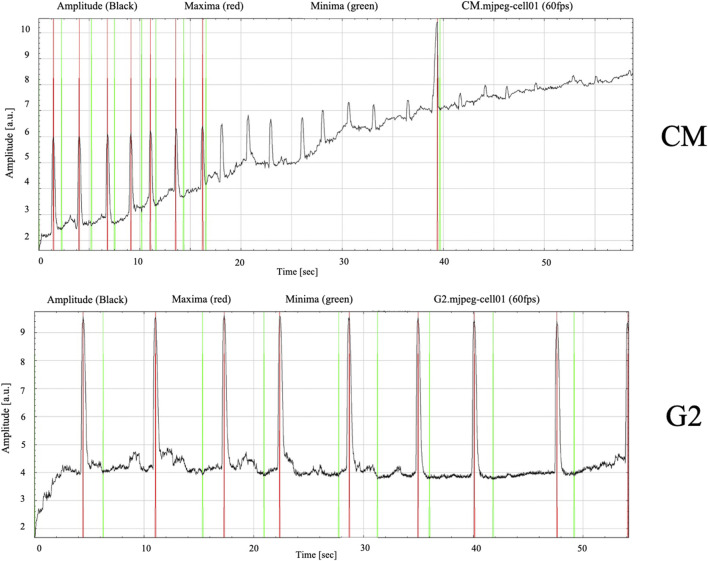
Contracting frequency of cardiomyocytes measured over a 60-s recording period. Images obtained from MYOCYTER software as dataplot.

Results related to contraction analysis are shown in Table 1 and Figure 10. These analyses revealed that, in presence of G2, the overall contraction phase (peaktime user) takes shorter time compared to the control. Moreover, the presence of G2 induce significantly shorter systolic contractions (contraction phase) with respect to the control, as well as in diastolic relaxation (relaxing time), indicating a shift in the contraction-relaxation phases, which may enhance the cardiomyocyte contractile efficiency.

**TABLE 1:** Contraction analysis data from MYOCYTER software. Data represent the results from three different experiments, expressed as mean ± SD.

Conditions	Frequency [1/sec]	Amplitude [a.u.]	Systole user [sec]	Diastole user [sec]	Peak time user [sec]
CM	0.73 ± 1.3	4.33 ± 2.8	7.82 ± 4.4	5.29 ± 2.3	13.11 ± 3.7
CM G2	1.51 ± 1.6	8.19 ± 1.4	0.42 ± 0.3*	0.57 ± 0.7*	1.00 ± 0.4 *

*Statistical significance with respect to control with p ≤ 0.05.

Finally, the amplitude, which is the contraction strength in arbitrary units, and the frequency seemed to be guided by the presence of the peptide, confirming the role of G2 in cardiac contractile phenotype maintenance. Figure 10 show how the contraction dynamics, with red signals representing high-intensity contraction peaks (maximum amplitude), and green signals reflecting baseline rhythm. In the control cells, contractions appeared irregular and uncoordinated, as evidenced by peak variable in amplitudes and inconsistent intervals between peaks, reflecting an unsynchronous contractile behavior. Meanwhile, in presence of G2, the graph shows relatively regular beating frequency throughout the measured period, suggesting improved electrical and mechanical synchronization.

## 4 Discussion

Cardiac ECM is necessary for cell migration, proliferation, and differentiation and is important for the structural integrity and elasticity of heart tissue, providing mechanical stiffness (Bildyug, 2019). Moreover, the ECM has a pivotal role in cardiomyogenesis, thus, some methods of cardiogenic differentiation include ECM proteins as a scaffold to enhance differentiation of cells towards CMs. Due to their specificity, high stability, and ability to be easily tailored and immobilized, ECM-related peptides are considered potent recombinant protein alternatives (Abdal Dayem et al., 2023). Moreover, peptides can emulate the functions of their full-length native counterparts.

Among all the components of ECM, laminins, especially laminin-221, represent a major component in the cardiac extracellular environment. Laminins are heterotrimeric basement membrane glycoproteins composed of α, β, and γ chains. Many active sequences have been found in the laminin globular domain (LG domain) at the C-terminal region of the α chain and shown to play an essential role in the interactions with cell surface receptors in a peptide and cell type-specific manner. The expression of the laminin α2 chain is crucial for muscle formation and regeneration (Wang et al., 2019; Barraza-Flores et al., 2020).

In order to define the adequate concentration of the laminin-derived peptide, we at first evaluated in both cells the cytoskeletal rearrangements induced by integrin-peptide interaction. The sarcomeric cytoskeleton represents the main structural element of myocytes essential in the regulation of cell shape and mechanical integrity, providing the uniform transmission of tension along myofibrils (Casarella et al., 2024; Zamir and Geiger, 2001; Burridge and Fath, 1989; Legerstee and Houtsmuller, 2021). We demonstrated that the presence of the peptide led to the structural reorganization of actin stress fibers, which represent a specialized form of F-actin associated with myosin II filaments, crosslinked by alpha-actinin and other associated proteins (e.g., vimentin, zyxin, and FA complex) (Casarella et al., 2024; Legerstee and Houtsmuller, 2021).

Additionally, the cytoskeleton allows an integrated connection to the extracellular matrix through the FAs, which mediate cell adhesion by connecting the cytoskeleton to the extracellular matrix, allowing the muscle to sense and respond to biomechanical stress (Zamir and Geiger, 2001; Sequeira et al., 2014). In a previous work, we demonstrated the role of vinculin and FAs in cardiac differentiation (Carton et al., 2023), here we evaluated the FAs formation in hMSC-Y201 and primary cardiac cells, demonstrating how the G2 peptide enhanced the FAs in differentiated hMSC-Y201. Meanwhile, in primary cardiac cells, the FAs resulted comparable in respect to the control when in presence of G2 at a lower concentration, suggesting that, when in presence of primary/differentiated cells, G2 can maintain the physiological and morphological state of actin fibers and FAs. In fact, it has been proved that laminin-221, by binding specifically to integrin α7β1, plays a crucial role in the enhancement of differentiation, growth, and viability of hiPS‐CMs and hESC (Samura et al., 2020; Yap et al., 2019; Nishiuchi et al., 2006).

Cardiac gene expression revealed the ability of the peptide to support cardiac differentiation, enhancing, in MSC, the expression of GATA-4 and NKX 2.5 genes, a cardiac transcription factor that has been shown to play critical roles in development, regulation of differentiation and control of cell proliferation and movement (Durocher et al., 1997; Saadane et al., 1999; Sepulveda et al., 1998). Interestingly, gene expression of GATA-4 in primary cardiomyocytes resulted constant in G2 compared to the control, indicating the ability of the peptide in supporting cardiac phenotype maintenance.

Moreover, the protein expression revealed that connexin-43 was upregulated in both cells when in presence of G2, indicating the pivotal role in guiding cytoskeletal changes and regulating the contractile activity. Connexins play a crucial role in forming gap junctions (GJs) at cardiac intercalated discs, which allow cell-cell communications and coupling electrical activity. Literature reports that a downregulation of connexin-43 is associated with excessive fibrotic deposition, ventricular dysfunction and weakened GJs (Jansen et al., 2017; Liu et al., 2022; Rodríguez-Sinovas et al., 2021). Intriguingly, primary cardiomyocytes, when in presence of G2 at 50 μg/mL, showed enhanced contractility and promoted cell clustering, allowing for more synchronized beating with improved force transmission during contractions.

It is well known that laminins, by interacting with integrins, can modulate the mechanotransduction and regulate costameric organization (Casarella et al., 2024). In addition, mutations in the integrin α7 gene are associated with adult‐onset cardiac dysfunction in humans and mice, and connexin-43 redistribution might be associated with the mechanosensing and mechanotransduction function of the integrin α7 in cardiac muscle (Okada et al., 2013; Bugiardini et al., 2022; Israeli-Rosenberg et al., 2014). Integrin α7β1 is also expressed in satellite cells and differentiating myoblasts and serves as an important regulator of muscle regeneration and repair (Bugiardini et al., 2022).

We found that laminin-derived peptide enhanced the expression of the two subunits of integrin, and when hMSC-Y201 was cultured with the neutralizing antibody anti-integrin β1, they loss the cytoskeletal rearrangement in differentiated cells. Moreover, the cardiac differentiation gene NKX 2.5 was downregulated. These data are consistent with previous work in which integrins-related early regulation of cardiomyocyte behavior and organization has been demonstrated (Miao et al., 2024). The combined expression of α and β integrin subunits is needed for expression of integrin receptors on the cardiomyocytes membrane, and the loss of β1 prevents cardiomyocytes from engaging the ECM network, resulting in failure to establish tissue architecture (Okada et al., 2013; Miao et al., 2024). However, due to the intrinsic expression in MSC of GATA-4, which ensures their maintenance of the multipotent differentiation capacity and proliferative potential (Babu et al., 2025), the differentiated cells demonstrated to still have an enhanced gene expression, even in presence of the neutralizing antibody anti-integrin β1, possibly due to the differentiative factors that can influence the differentiation activating alternative pathways non-laminin-dependent.

The present study demonstrated how laminin-derived peptide enhances cardiac differentiation of MSCs and influence cell behavior of primary cardiomyocytes, driving and maintaining the contractile phenotype.

Thus, given their role in guiding cardiomyogenesis, in order to restore the cardiac phenotype and prevent tissue necrosis after MI, promoting the cell-matrix interactions and cell adhesion, the active-peptides derived from laminin-2 may be used to functionalize scaffolds and matrices, such as hydrogels, for regenerative purposes. However, this strategy could face limitations in the stability of the peptides, potentially affecting the release in terms of therapeutic efficacy and *in vivo* degradation of the matrix. Moreover, functionalization of the matrices with short peptides may require additional molecules in order to achieve the optimal mechanical properties, while affecting the biological efficacy. Ongoing research and technological advancements in this field hold significant promise for enhancing treatment outcomes for cardiac damage and other related disorders. By exploiting these innovative regenerative approaches, the objective of restoring functional cardiac muscle tissue after MI and improving the quality of life for affected individuals is becoming increasingly reachable.

## Data Availability

The raw data supporting the conclusions of this article will be made available by the authors, without undue reservation.
