# Supplementary material for: Laminin-derived peptide drives the cardiomyogenic potential and cardiac cells functionality
Source: Front Bioeng Biotechnol. 2025 Aug 8;13:1629412. doi: 10.3389/fbioe.2025.1629412 (PMC12370699; doi:10.3389/fbioe.2025.1629412)
Supplement: Supplementary file 1 [file Supplementaryfile1.docx]

Supplementary Material

Laminin-derived peptide drives the cardiomyogenic potential and cardiac cells functionality

Simona Casarella^1^, Federica Ferla^1^, Dalila Di Francesco^1,2^, Veronica Pagani^1^, Carolina Di Varsavia^1^, Irene Regano^1^, Francesca Boccafoschi^1,*^

^1^Department of Health Sciences, University of Piemonte Orientale “A. Avogadro”, Novara, Italy

^2^Laboratory for Biomaterials and Bioengineering, Canada Research Chair Tier I for the Innovation in Surgery, Department of Min-Met-Materials Engineering and Regenerative Medicine, CHU de Quebec Research Center, Laval University, Quebec City, QC, Canada

*** Correspondence:**Corresponding Author
francesca.boccafoschi@med.uniupo.it

# Supplementary Material

**Supplementary Table 1: Forward and reverse human primers sequences**

| RNA | Forward primer | Reverse primer |
| --- | --- | --- |
| GATA-4 | 5’-TCCAAACCAGAAAACGGAAG-3’ | 5’-CTGTGCCCGTAGTGAGATGA-3’ |
| TROPONIN I | 5’-GGACAAGGTGGATGAAGAGA -3’ | 5’-AGGGTGGGCCGCTTAAACT-3’ |
| NKX 2.5 | 5’-ACCTCAACAGCTCCCTGACTC-3’ | 5’-ATAATCGCCGCCACAAACTCTCC-3’ |
| GAPDH | 5’-GTATGACAACAGCCTCAAGAT-3’ | 5’-GTCCTTCCACGATACCAAAG-3’ |

**Supplementary Table 2: Forward and reverse mouse primers sequences**

| RNA | Forward primer | Reverse primer |
| --- | --- | --- |
| GATA-4 | 5’-TCCAGTGCTGTCTGCTCTAAGC-3’ | 5’-TGGCCTGCGATGTCTGAGT-3’ |
| TROPONIN I | 5’-TCTGCCAACTACCGAGCCTAT-3’ | 5’-CTCTTCTGCCTCTCGTTCCAT-3’ |
| DESMIN | 5’-GTGGATGCAGCCACTCTAGC -3’ | 5’-TTAGCCGCGATGGTCTCATAC-3’ |
| GAPDH | 5’-CGACCCCTTCATTGACCTCAAC-3’ | 5’-CTCCACGACATACTCAGCACC-3’ |

# Supplementary Data

**Supplementary Video 1 (CM).** Video recording of beating cluster of CM in control condition. Video was recorded using a brightfield Optical Microscopy and captured at 60 frame rates of 60 seconds using a camera attached to the microscope ocular with a universal adapter. Scale Bar: 100 µm.

**Supplementary Video 2 (G2).** Video recording of beating cluster of CM with G2. Video was recorded using a brightfield Optical Microscopy and captured at 60 frame rates of 60 seconds using a camera attached to the microscope ocular with a universal adapter. Scale Bar: 100 µm.
